# Dynamic nature of SecA and its associated proteins in *Escherichia coli*

**DOI:** 10.3389/fmicb.2015.00075

**Published:** 2015-02-10

**Authors:** Shun Adachi, Yasuhiro Murakawa, Sota Hiraga

**Affiliations:** Department of Radiation Genetics, Graduate School of Medicine, Kyoto University Kyoto, Japan

**Keywords:** chromosome partition, SecA, SecY, AcpP, MukB, DNA topoisomerase

## Abstract

Mechanical properties such as physical constraint and pushing of chromosomes are thought to be important for chromosome segregation in *Escherichia coli* and it could be mediated by a hypothetical molecular “tether.” However, the actual tether that mediates these features is not known. We previously described that SecA (Secretory A) and Secretory Y (SecY), components of the membrane protein translocation machinery, and AcpP (Acyl carrier protein P) were involved in chromosome segregation and homeostasis of DNA topology. In the present work, we performed three-dimensional deconvolution of microscopic images and time-lapse experiments of these proteins together with MukB and DNA topoisomerases, and found that these proteins embraced the structures of tortuous nucleoids with condensed regions. Notably, SecA, SecY, and AcpP dynamically localized in cells, which was interdependent on each other requiring the ATPase activity of SecA. Our findings imply that the membrane protein translocation machinery plays a role in the maintenance of proper chromosome partitioning, possibly through “tethering” of MukB [a functional homolog of structural maintenance of chromosomes (SMC) proteins], DNA gyrase, DNA topoisomerase IV, and SeqA (Sequestration A).

## Introduction

In eukaryotes, mitotic spindles attach to kinetochores of chromosomes to assure bi-oriental segregation of sister chromosomes into the two dividing daughter cells. In prokaryotes, however, no such structure has been identified, and the mechanisms mediating faithful chromosome segregation in bacteria remain elusive. On the one hand, Jun and Mulder (2006) proposed entropic forces as driving forces for compartmentalization of polymeric sister chromosomes in the cylindrical cell periphery, a concept that has been confounded by a number of discrepancies (Odijk, 1998; Cunha et al., 2001; Wiggins et al., 2010; Hadizadeh Yazdi et al., 2012; Fisher et al., 2013). On the other hand, Bates and Kleckner (2005) proposed that the principal mechanism of chromosome segregation involves a mechanical pushing force from coherent chromosomes followed by the release from a tether. These models raise the question of whether the nature of the energy required for segregation depends on passive entropic forces or active mechanisms involving ATPases. Recently, four-dimensional imaging of the *Escherichia coli* nucleoid revealed surprising dynamics such as individualization, radial confinement, and longitudinal density spots moving back and forth that can affect segregation of tortuously structured sister chromosomes (Fisher et al., 2013). More specifically, (I) sister chromosomes occupy their respective spaces due to initial bundling of chromosomes; (II) molecular tethers minimize radial confinement of chromosomes; (III) stressing and releasing cycles of this “tether” allow chromosomes with longitudinal density spots to move back and forth, acting as “grease.” Bundling, confinement, and stressing of chromosomes are all mediated by this “tether,” followed by release from the same “tether,” illustrating the mechanical forces required for chromosome segregation. Therefore, progression through the steps of chromosome segregation may involve cyclic accumulation and release of intranucleoid mechanical stress mediated by the hypothetical molecular “tether.” However, the actual molecular “tether” has not been identified.

One of the clues for identifying the actual molecular tether is that the molecular tether is likely to be one or more proteins involved in chromosome positioning and partitioning. In addition, the molecular tether may also show dynamic localization as is seen with the nucleoid. In *E. coli*, MukB (“mukaku” means anucleate in Japanese) (Niki et al., 1992) is a functional homolog of SMC (structural maintenance of chromosomes) and is involved in activities of chromosome organization such as positioning and partitioning, i.e., proper localization of the *oriC* region, nucleoids, and replication forks. MukB localizes equidistantly as clusters along the long cell axis in living cells (Hiraga, 2000; Ohsumi et al., 2001; Adachi et al., 2008; Nolivos and Sherratt, 2014). Similarly, SeqA (Sequestration A) also appears to be spatially regulated. SeqA foci localize equidistantly along the long cell axis, and this ordered localization is hypothesized to be important for chromosome organization (Hiraga et al., 1998, 2000; Onogi et al., 2002; Yamazoe et al., 2005). These proteins might interact with molecular tether. Furthermore, MukB molecules form one to three foci in a living cell together with other mobile MukB molecules that show dynamic localization, suggesting roles for MukB in chromosome positioning/partitioning and/or condensation (Badrinarayanan et al., 2012). The dynamics of MukB thus indicates its interaction with molecular tether.

SecA (Secretory A) plays a role in the translocation of membrane proteins through attaching to and dismantling from the Sec pore complex of the inner cell membrane, shuttling between inner cell membrane and cytosol, thus maintaining homeostatic conditions in cells via membrane transport machinery (Cabelli et al., 1991; Robson and Collinson, 2006). Secretory Y (SecY) is a component of the membrane protein translocation channel (Robson and Collinson, 2006). SecY and SecB interact with SecA and stimulate the ATPase activity of SecA (Miller et al., 2002; Natale et al., 2004). Regarding the function of the ATPase of SecA, “Push and Slide” mechanism, in which translocating peptide is pushed toward outer direction during ATP-bound phase in immobile form, followed by ATP hydrolysis and investing sliding mobility to the peptide. Finally, ADP is converted to ATP and the cycle is repeated again and again (Bauer et al., 2014). Previously we have described that SecA, SecY, and Acyl carrier protein P (AcpP) are involved in chromosome partition and that inactivation of SecA causes disorder in decatenation and maintenance of superhelicity on plasmid DNA (Adachi et al., 2014). AcpP, which is an acyl carrier protein of ACP complex, acts as a source of acyl groups inside the cell of *E. coli*. Acylated proteins are transferred to membrane by lipid-acyl group interaction. For example, bacterial toxin Haemolysin is distributed to cell membrane by this process (Issartel et al., 1991; also refer Towler and Gordon, 1988; Cronan and Rock, 1996). Therefore, it can be speculated that AcpP relates protein dynamics closely related to the inner cell membrane. Furthermore, AcpP is copurified with MukB (Niki et al., 1992), suggesting the role of AcpP on chromosome segregation. It is notable that AcpP binds to both SecA and MukB, In addition, we have described that a network involving the interaction of SecA/SecY-AcpP-MukB-Topo IV (DNA topoisomerase IV, ParC/ParE complex)/SeqA-DNA gyrase (GyrA/GyrB complex) may be important for chromosome positioning and partitioning (separation of chromosomes) (Adachi et al., 2014).

In the present work, we examined subcellular localization of these proteins and found that these proteins trace tortuous nucleoids, and were dynamic. The dynamic localization of SecA was ceased by inactivation of SecY and AcpP. Conversely, dynamic localization of SecY was ceased by inactivation of SecA. We revealed that the ATPase activity of SecA was essential for dynamic localization of these proteins. Integrating the present results with our previous work that SecA/SecY/AcpP genetically interact with MukB and DNA topoisomerases (Adachi et al., 2014), we hypothesize that SecA/SecY/AcpP is the core component of the dynamic property of the chromosome segregation proteins in *E. coli*.

## Materials and methods

### Bacterial strains and plasmids

The strains and plasmids used in this study are shown in Tables S1 and S2, respectively. Derivatives with the azide-resistant *secA204* mutation were obtained by P1 transduction (using P1_*vir*_) of W208 and selection and purification with 3–4 mM sodium azide. Derivatives with the *mukB-gfpUV4* mutation were obtained by co-transduction of KAT1 with *cat* and selection and purification with 5 μg/ml chloramphenicol. *Fab*^+^ derivatives of the NAD strains were obtained by co-transduction of TL212 with *fab*^+^ and the *zce-726*::*Tn*10 marker from TL212 and selection and purification with 7.5 μg/ml tetracycline. We also created the MQ574 strain by P1_*vir*_ transduction of JC12334 with the *tna-300*::*Tn*10 mutation [linked to *gyrB*(Ts)] and selection and purification with 7.5 μg/ml tetracycline. Derivatives with the *gyrB*(Ts) mutation were obtained by P1_*vir*_ transduction of MQ574.

### Media

Medium L (Hiraga et al., 1989) consists of 1% Bactotryptone (Difco, Sparks, MD), 0.5% yeast extract (Difco), and 0.5% NaCl (pH 7.4). Medium C consists of synthetic medium M9 (Miller, 1992) supplemented with 0.5% glycerol, 50 μg/ml l-threonine, l-leucine, l-proline, l-arginine, l-histidine, l-tryptophan, and 5 μg/ml vitamin B1. l-Glutamate (50 μg/ml) and chloramphenicol (5 μg/ml) were added to the cultures of strains when necessary.

### Immunofluorescence microscopy

Indirect immunofluorescence microscopy of *E. coli* cells was performed as described previously (Hiraga et al., 1998; Hirano et al., 1998). Rabbit anti-SecA/SecY antisera were gifts from Dr. Koreaki Ito (Kyoto Sangyo University). Rabbit anti-AcpP antiserum was a gift from Dr. Charles Rock (St. Jude Children's Research Hospital). Mouse anti-GFP monoclonal antibody is commercially available (Roche Diagnostics, K.K., Tokyo, Japan). We used goat anti-rabbit/mouse IgG antiserum conjugated to Cy3 (GE Healthcare, U.K. Ltd., Little Chalfont, UK) as the secondary antibody. For optical sectioning, an OLYMPUS BX61 microscope with an OLYMPUS UPlanApo ×100/1.35 oil objective lens (OLYMPUS, Corp., Tokyo, Japan) connected to CoolSNAPHQ (NIPPON ROPER, K.K., Chiba, Japan) was equipped with MetaMorph (Universal Imaging, Corp., Downingtown, PA) to obtain series of Z-sections with a fixed spacing of 0.1 μm. Each stack of 26 optically sectioned fluorescent images was deconvoluted through 50 iterations using the three-dimensional deconvolution function in the AutoQuant X version 1.4.1 program (Media Cybernetics, Inc., Silver Spring, MD). Three-dimensional images were then obtained by Volume Viewer function of 3D plug-in in ImageJ software.

### Time-lapse experiments for GFP_UV4_-fused proteins in living cells

We used cultures grown in medium C at 30°C unless otherwise indicated. IPTG (isopropyl ß-d-1-thiogalactopyranoside, 0.1 mM) was added and incubated for 1 h when we observed a GFP_uv4_ (uv4 is a GFP variant with strong fluorescence and an altered spectrum; Ito et al., 1999) signal in living cells. Ten microliters of the cell suspension was fixed on a 1% agarose/medium-coated glass slide, covered with a cover glass, and sealed with vacuum grease. Images of the fluorescence signal were observed using an OLYMPUS BX51 fluorescence microscope with an OLYMPUS UApo ×2×150/1.45 oil objective lens (OLYMPUS, Corp.) connected to Cascade II 512 (Photometrics, Tucson, AZ) equipped with MetaMorph (Universal Imaging, Corp.). The obtained fluorescence signals were averaged over cell width and plotted as histograms along long cell axes by ImageJ. All time-lapse images were exposed for 100 ms to capture the indicated images of **Figure 2** with 45-s intervals for approximately 3 min or of Supplementary Movies with 3 s/frame for approximately 5 min.

### Conventional fluorescence microscopy

Microscopy was performed as described in Adachi et al. (2008).

### Run-off replication method to analyze the DNA content per cell using flow cytometry

The procedure was performed as previously described (Adachi et al., 2008).

### FRAP (fluorescence recovery after photobleaching) microscopy

Images of fluorescence signals were observed using an OLYMPUS Fluoview™ FV1000 confocal fluorescence microscope connected to OLYMPUS IX81 with an OLYMPUS PlanSApo ×60/1.35 oil objective lens (OLYMPUS, Corp.) in a pre-warmed chamber. The images were taken with FLUOVIEW Ver. 1.6 software and were simultaneously subjected twice to Kalman line function. For setting up the conditions, see Supplementary Text.

## Results

### Subcellular localization of SecA, SecY, and AcpP proteins embracing nucleoids

We used PA340 cells expressing proteins fused to GFP_uv4_. The GFP_uv4_-fused proteins are inducible by 0.1 mM IPTG. All strains that harbored plasmids carrying genes encoding GFP_uv4_-fused proteins grew normally and similarly and the localizations of GFP_uv4_-fused proteins were indistinguishable in the presence or absence (under leaky expression status) of 0.1 mM IPTG, suggesting that IPTG induction of GFP_uv4_-fused proteins did not affect localization of protein molecules or cell growth. Importantly, we confirmed that all the GFP_uv4_-fused proteins described in this manuscript corrected the defects of the corresponding temperature sensitive or deletion mutations in *E. coli*, showing that these fusion proteins were functional.

**Figure 1:**
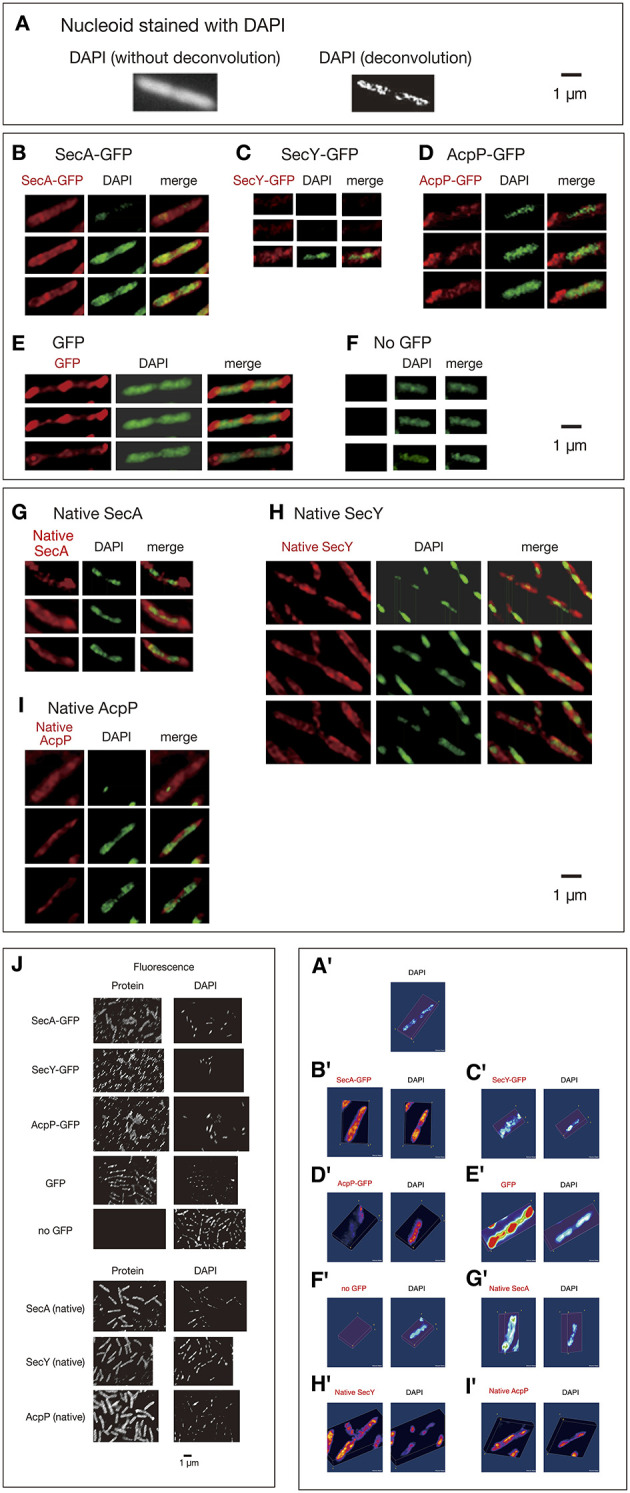
**Three-dimensional deconvoluted images of various immunostained proteins**. Cells were grown in medium L at 30°C and fixed with 70% methanol. Red, immunofluorescence of GFP or native proteins. Green, DAPI staining. Merge, merged images of red and green images. Note that synthesized yellow signal is only recognizable with eyes when the ratio of each signal is between ~4:6 and 6:4. More difference is recognizable as mere green or red. **(A)** Images of a DAPI-stained wild-type cell before and after 3D deconvolution in the Z-axis. **(B–F)** Fixed cells expressing GFP_uv4_-fused proteins or GFP_uv4_ protein (not fused with any protein) were immunostained using an anti-GFP monoclonal antibody. Fluorescence images of the proteins were analyzed with 3D deconvolution at 0.5-μm intervals in the Z-axis. Images of DAPI-stained nucleoids were also analyzed with 3D deconvolution. The yellow arrowheads indicate the clustered localization of protein molecules. **(B)** SecA-GFP_uv4_, **(C)** SecY-GFP_uv4_, **(D)** AcpP-GFP_uv4_, **(E)** GFP_uv4_ (not fused with any protein), **(F)** No GFP_uv4_. **(G–I)** Deconvoluted images of native proteins. Wild-type cells (PA340) were grown in medium L at 37°C, fixed, and immunostained using rabbit antisera for native proteins. Images of DAPI-stained nucleoids were also deconvoluted. **(G)** Native SecA, **(H)** Native SecY, **(I)** Native AcpP, **(J)** Three-dimensional deconvolution images of immunostained cells in wider sights corresponding to **(B)**–**(I)**. (**A'–I'**) Three-dimensional images corresponding to **(A)**–**(I)**.

Cells grown in rich medium L were fixed and stained for immunofluorescence using anti-GFP monoclonal antibody. To obtain images with high-resolution without out-of-focus fluorescence, methanol-fixed and immunostained cells were analyzed by three-dimensional deconvolution. Imaging of living cells is not feasible for 3D deconvolution, because of rapid movements of protein molecules within the time range of obtaining Z-stacked images as described in the following section. First, 3D deconvolution of DAPI (4,6′-diamidino-2-phenylindole)-stained cells showed that nucleoids exhibited a tortuous structure (Figures 1A,A',B,B'; Movies S1, S3) as reported previously by Hadizadeh Yazdi et al. (2012) and Fisher et al. (2013). We next examined the subcellular localization of SecA-GFP_uv4_. Three-dimensional Deconvolution of SecA-GFP_uv4_ images revealed that the protein was localized with condensed regions tracing over, complementing or in parallel with nucleoid structures (Figures 1B,B'; Movies S2–S4). Although the condensed regions of SecA-GFP_uv4_ overlapped with the nucleoid, patterns of the condensed regions were not entirely the same as those of nucleoids (Figures 1B,B'; Movies S2, S4). Furthermore, SecY-GFP_uv4_ (Figures 1C,C'; Movies S5–S7) and AcpP-GFP_uv4_ (Figures 1D,D'; Movies S8–S10) were also localized as tortuous structures embracing nucleoids with asymmetric condensed regions. However, the patterns of the condensed regions were not entirely the same as those of the nucleoids. In contrast, GFP_uv4_ (not fused to a protein) was intensively localized in cytosolic areas with absolute symmetry and was faint in regions occupied by nucleoids apparently different from more uniform distribution without deconvolution (Figures 1E,E'; Movies S11–S13). This is, to our knowledge, the first deconvolved image of non-fused GFP in *E. coli*, which are supposed to reflect more precise information, such as exclusion of GFP_uv4_ from tightly packed nucleoid. As a control, cells without GFP_uv4_ did not exhibit a fluorescence signal (Figures 1F,F', Movies S14, S15).

Additionally, we confirmed that the native SecA, SecY, and AcpP proteins without GFP_uv4_, which were immunostained with specific rabbit antisera for each protein, were also localized as asymmetric tortuous structures with condensed regions complementing or in parallel with nucleoids similar to the tagged proteins (Figures 1G,G',H,H',I,I'; Movies S16–S24). Although in Swulius and Jensen (2012) artificial helices of YFP-tagged MreB was reported, our results for native antisera suggest that the localization of GFP_uv4_-fused proteins was not an artifact due to GFP_uv4_ tagging. In all of the images, the localizations of the proteins extended out of nucleoids regions, including polar localization. Cells of wider sights are shown in Figure 1J.

### Dynamic localizations of SecA, SecY, and other proteins in living cells

**Figure 2:**
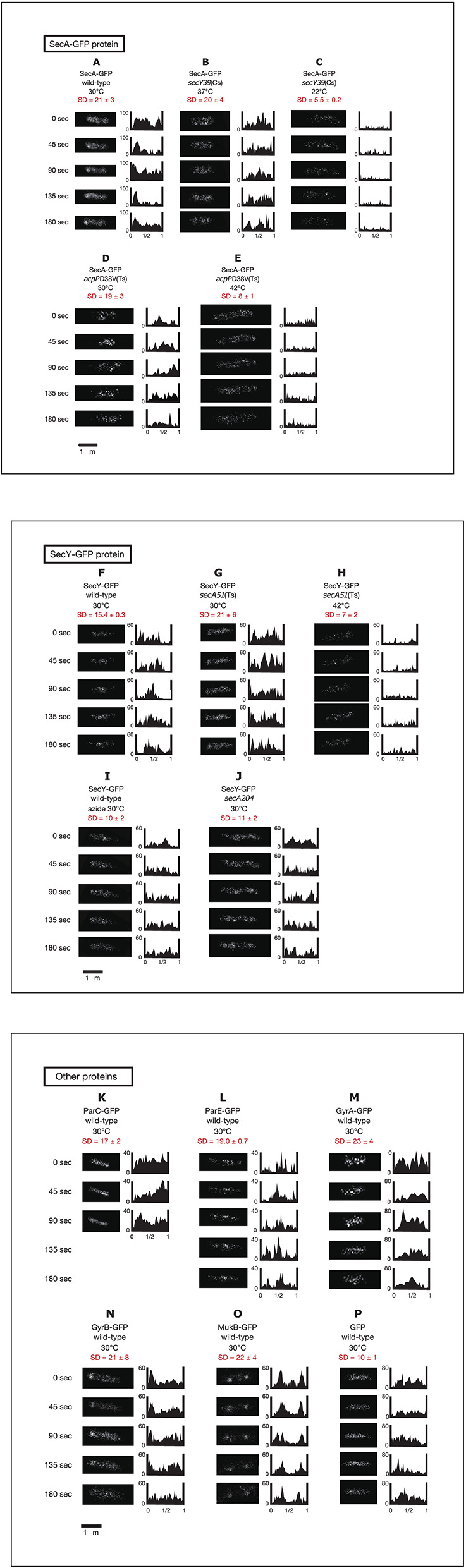
**Time-lapse images of GFP_uv4_-fused proteins reveal the dynamic nature of chromosome partitioning proteins**. Cells were grown in medium C and incubated with 0.1 mM IPTG for 1 h. Time-lapse images of fluorescence were taken by exposure for 100 ms. Both images and histograms of signals along long cell axes were obtained at 45-s intervals. The x-axes of histograms are subcellular positions and the y-axes of histograms are relative fluorescence units. Ninety-five Percent confidential SD (standard deviation) values of fluorescence signals along long cell axes in individual cells are also shown. **(A)** SecA-GFP_uv4_ in wild-type cells (MQ318) growing at 30°C. **(B)** SecA-GFP_uv4_ in *secY39*(Cs) mutant cells (MQ435) growing at the permissive temperature of 37°C. **(C)** SecA-GFP_uv4_ in *secY39*(Cs) mutant cells (MQ435) growing at the non-permissive temperature of 22°C. **(D)** SecA-GFP_uv4_ in *acpP*D38V(Ts) mutant cells (MQ456) growing at the permissive temperature of 30°C. **(E)** SecA-GFP_uv4_ in *acpP*D38V(Ts) mutant cells (MQ456) growing at the non-permissive temperature of 42°C. **(F)** SecY-GFP_uv4_ in wild-type cells (MQ319) growing at 30°C. **(G)** SecY-GFP_uv4_ in *secA51*(Ts) mutant cells (MQ748) growing at the permissive temperature of 30°C. **(H)** SecY-GFP_uv4_ in *secA51*(Ts) mutant cells (MQ748) growing at the non-permissive temperature of 42°C. **(I)** SecY-GFP_uv4_ in wild-type cells (MQ319) with 1 mM sodium azide growing at 30°C. The incubation time with azide was approximately 5 min. **(J)** SecY-GFP_uv4_ in *secA204* azide-resistant mutant cells (MQ625) growing at 30°C (without sodium azide). **(K)** ParC-GFP_uv4_ in wild-type cells (MQ537) growing at 30°C. **(L)** ParE-GFP_uv4_ in wild-type cells (MQ539) growing at 30°C. **(M)** GyrA-GFP_uv4_ in wild-type cells (MQ323) growing at 30°C. **(N)** GyrB-GFP_uv4_ in wild-type cells (MQ324) growing at 30°C. **(O)** MukB-GFP_uv4_ in wild-type cells (MQ529) growing at 30°C. **(P)** GFP_uv4_ in wild-type cells (MQ668) growing at 30°C.

We next performed time-lapse experiments to analyze living cells expressing GFP_uv4_-fused proteins growing in poor medium C because the number of condensed regions in cells expressing GFP_uv4_-fused proteins was lower in poor medium than in rich medium, allowing easier study of the dynamic behavior of the proteins. GFP_uv4_-fused proteins were induced by incubation with 0.1 mM IPTG for 1 h. Cells were photographed with a short exposure (100 ms) with some of the fluorescence signals. In this exposure, especially highly mobile diffusing molecules were unable to be detected. The fluorescence signals obtained were plotted as histograms at 45-s intervals for approximately 3 min. To monitor rapid movements of protein molecules, obtaining Z-stacked images, which were necessary for three-dimensional deconvolution in living cells, was not feasible. In addition, obtaining phase-contrast images side by side with time-lapse images was not possible because filter exchange was not practical with such high-speed imaging. The localization of fluorescence signals is evaluated by standard deviation (SD) values of relative signal values along long cell axes of 2–12 cells. If the SD value is larger, non-uniform localization of the signals is expected. If smaller, spatially rather uniform signals are expected. The results are summarized in Figure 2.

Surprisingly, GFP_uv4_-fused SecA protein molecules showed dynamic localization in living cells (Movie S25). SecA-GFP_uv4_ was observed as clusters that repeatedly accumulated and dissociated at one cell pole or the other, or at other cellular positions as density spots moving back and forth (Figure 2A; Movie S25). This is consistent with the result that SecA is present in both peripheral region of cell membrane and cytoplasm. SecY-GFP_uv4_ behaved in a similar manner as SecA-GFP_uv4_, with slightly more uniform distribution (Figure 2F; Movie S30).

**Figure 3:**
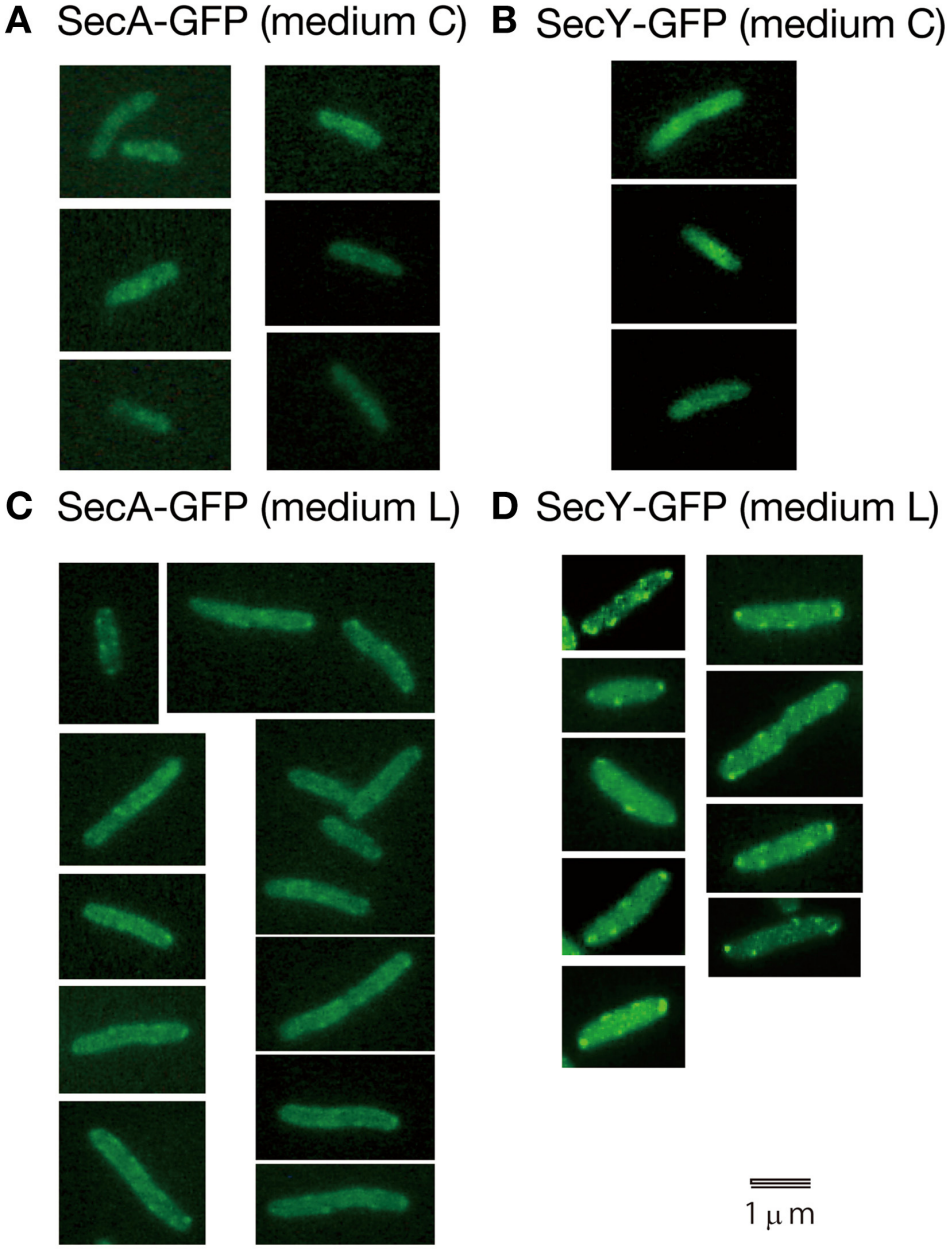
**Living wild-type cells with SecA-GFP_uv4_/SecY-GFP_uv4_ were observed with a conventional fluorescence microscope. (A)** Fluorescence signal of SecA-GFP_uv4_ in cells grown in medium C. **(B)** Fluorescence signal of SecY-GFP_uv4_ in cells grown in medium C. **(C)** Fluorescence signal of SecA-GFP_uv4_ in cells grown in medium L. **(D)** Fluorescence signal of SecY-GFP_uv4_ in cells grown in medium L.

Living cells with SecY-GFP_uv4_ grown in media C and L were observed with a conventional fluorescence microscope (Figure 3). In living cells grown in richer medium L, strong fluorescence foci of SecY-GFP_uv4_ were observed (Figure 3D). The strong foci appeared to localize in peripheral regions of cell membrane. The foci might be assembled clusters of many membrane protein translocation channels. The strong fluorescence signal from SecY-GFP_uv4_ (and also SecA-GFP_uv4_) foci are prominent in Figures 3C,D. However, in immunostaining images of Figure 1, the contrast was not so prominent, despite there are still vague fluorescence peaks supposed to be originated from the former foci in living cells. During permeablization of immunostaining procedure, some of the proteins might be dissociated from the corresponding foci closely located near cell membrane.

ParC-GFP_uv4_ accumulated and dissociated in a similar manner to that of SecA-GFP_uv4_ (Figure 2K; Movie S35). ParE-GFP_uv4_ appeared to accumulate and dissociate within a near-polar or mid-cell region (Figure 2L; Movie S36). These results for ParC-GFP_uv4_ and ParE-GFP_uv4_ were consistent with previously reported results (Espeli et al., 2003).

GyrA and GyrB (subunits of DNA gyrase) fused to GFP_uv4_ showed dynamic localization in a similar manner as SecA-GFP_uv4_ (Figures 2M,N; Movies S37, S38). Previous transmission electron microscopy with immunogold particles indicates that Gyr protein molecules are predominantly localized at cytosol (~90%) mainly peripheral to nucleoids, suggesting their role in DNA segments unwinded from the nucleoid (Thronton et al., 1994). Since GFP would in principle localize outside the nucleoid with strong fluorescence from cell poles (Figure 1E), the localizations of Gyr proteins are different from GFP. This does not contradict with our analyses, since fluorescence signals all around the peripheral regions of nucleoid could result in broad signals that seem to surround the nucleoids, mimicking cytosol.

Live-cell imaging of MukB-GFP_uv4_ in strain MQ529 in which genomic *mukB* was replaced with *mukB-gfpUV4* (Ohsumi et al., 2001) showed preferential localization of MukB-GFP_uv4_ as two clusters in the cell quarter positions. An individual cluster showed frequent oscillation in which it divided into two or dispersed and fused again to form one cluster (Figure 2O; Movie S39).

AcpP-GFP_uv4_ exhibited tortuous localization with condensed regions or net-like localization, but its dynamics were not clear (data not shown). The localization patterns of these proteins were similar when concentrations of IPTG lower than 0.1 mM were used. Dynamic localization of these fusion proteins was observed at high frequencies as summarized in Table 1. As a control, GFP_uv4_ (not fused to a protein) was observed as dispersed signal peaks with no clusters distributed throughout the entire cell and did not exhibit clear dynamic localization (Figure 2P; Movie S40). Assuming that the observed proteins described in this section are more or less associated with nucleoid, these results are consistent with previous reports that described density spots moving back and forth in bacterial nucleoids (Fisher et al., 2013).

**Table 1:** **Frequencies of the observation of dynamic movement**.

SecA-GFP_uv4_	100% (97/97)
SecY-GFP_uv4_	99% (73/74)
AcpP-GFP_uv4_	53% (9/17)
ParC-GFP_uv4_	100% (6/6)
ParE-GFP_uv4_	91% (10/11)
GyrA-GFP_uv4_	100% (11/11)
GyrB-GFP_uv4_	100% (5/5)
MukB-GFP_uv4_	91% (72/79)

### Perturbation of the dynamic localization of SecY-GFP_UV4_ with the secA conditional mutation

We next examined effects of SecA on dynamic localization of SecY-GFP_uv4_. When temperature-sensitive *secA51* mutant cells (MQ748) were incubated at the non-permissive temperature of 42°C for 2 h, the dynamic localization of SecY-GFP_uv4_ was remarkably affected. Almost all the molecules seemed to scatter throughout the cell, moving very rapidly without localization (Figure 2H; Movie S32), in contrast to the dynamic clusters that were seen at the permissive temperature of 30°C (Figure 2G; Movie S31). As a negative control, the dynamic localization of SecY-GFP_uv4_ was not affected in isogenic wild-type *secA* cells (MQ743) by incubation at 42°C (data not shown). Thus, the dynamic localization of SecY-GFP_uv4_ depends on SecA function.

### Perturbation of the dynamics of SecA-GFP_UV4_ with secY and acpP conditional mutations

We also found that the dynamic localization of SecA-GFP_uv4_ was affected in cold-sensitive *secY39* mutant cells (MQ435) when incubated at the non-permissive temperature of 22°C for 5 min (Figure 2C; Movie S27), but not at the permissive temperature of 37°C (Figure 2B; Movie S26). In contrast, both dynamic localization of SecA-GFP_uv4_ was observed in the isogenic wild-type *secY* strain (MQ519) after incubation at 22°C (data not shown). These results show that SecY plays an important role in the dynamics of SecA-GFP_uv4_.

In addition, the dynamic localization of SecA-GFP_uv4_ was also affected in temperature-sensitive *acpP*D38V mutant cells (MQ456: amino acid substitution of V for D at amino acid position 38) at the non-permissive temperature of 42°C after incubation for 30 min (Figure 2E; Movie S29), but not at the permissive temperature of 30°C (Figure 2D; Movie S28). The fluorescence signal for SecA-GFP_uv4_ was observed even after incubation for 30 min at 42°C in the isogenic wild-type *acpP* strain (MQ516), suggesting that incubation at the higher temperature did not disrupt the dynamic localization of SecA-GFP_uv4_. Thus, the dynamic localization of SecA-GFP_uv4_ depends on the function of AcpP. Overall, these results demonstrate that the dynamic localization of SecA and SecY of the membrane protein translocation machinery and AcpP in cells was interdependent on each other.

### Effect of sodium azide on dynamic localization of SecY-GFP_UV4_

We next utilized 1 mM sodium azide to inhibit SecA ATPase activity. SecA, but not other essential ATPases, is considered to be the functional target of this concentration of sodium azide because the growth kinetics and cellular and nucleoid morphologies are normal in the azide-resistant *secA204* mutant (Adachi et al., 2014). The normal growth of the mutant indicates that respiratory metabolisms including cytochrome c oxidase activity are not significantly affected at this concentration. Perturbation of the dynamic localization of SecY-GFP_uv4_ was also observed in the presence of 1 mM sodium azide in wild-type strains (Figure 2I; Movie S33), indicating that the SecA ATPase activity is essential for the dynamics of these proteins. The dynamics of SecY-GFP was not clearly observed in the azide-resistant *secA204* mutant (Figure 2J; Movie S34) in contrast to the wild-type *secA* strain (Figure 2F; Movie S30), suggesting that this mutant was defective (or leaky) in the unknown function of SecA acting on SecY-GFP dynamics. This result was consistent with the result that the positioning of *oriC* was abnormal in the mutant (Adachi et al., 2014). The effect of azide was not observed in the *secA204* mutant (data not shown).

### Perturbations of the positionings of MukB and SeqA foci with sodium azide

**Figure 4:**
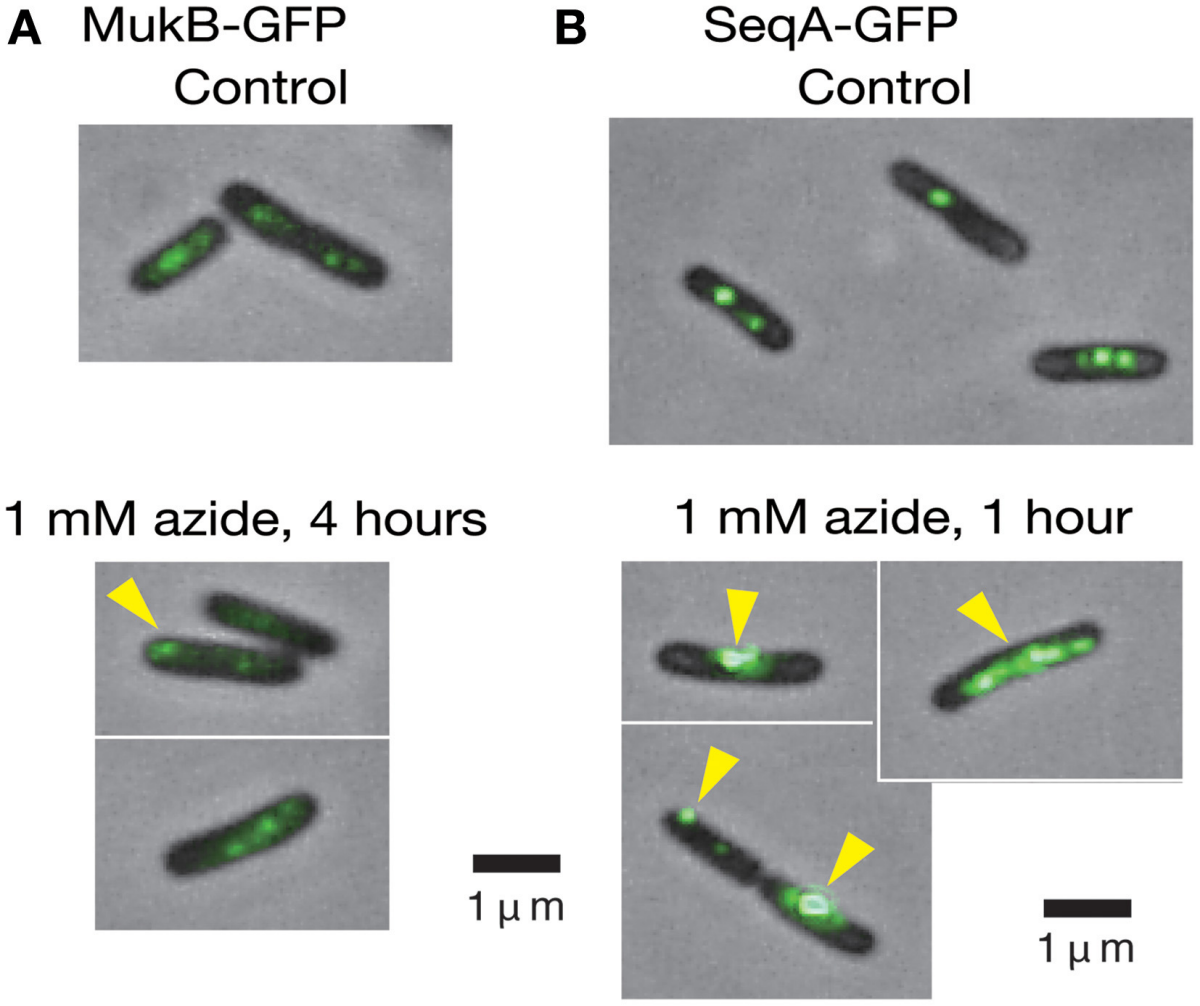
**Living cells with MukB-GFP_uv4_/SeqA-GFP_uv4_ were observed with a conventional fluorescence microscope. (A)** The effect of 1 mM sodium azide on the localization of MukB-GFP_uv4_ (MQ529). The yellow arrowhead shows abnormal localization of a fluorescent focus at the cell pole. **(B)** The effect of 1 mM sodium azide on localization of SeqA-GFP_uv4_ (MQ190). The yellow arrowheads show the abnormal localization of fluorescent foci at the cell pole and large fluorescent clusters.

Because 1 mM sodium azide perturbs the proper positioning of the *oriC* region (Adachi et al., 2014), we examined whether the compound has similar effects on the positioning of MukB, a key protein in the positioning of *oriC*. MukB-GFP_uv4_ foci in the *mukB-gfpUV4* strain (MQ525) are normally located at the mid-cell or cell quarter positions, implying a role for MukB in the chromosome reorganizing center by acting on condensation and proper patterning of the entire nucleoid (Ohsumi et al., 2001; Adachi et al., 2008). When the *mukB-gfpUV4* cells (MQ525) were treated with 1 mM sodium azide in medium C for 4 h, abnormal localization of MukB-GFP_uv4_ foci at cell pole(s) was observed in approximately 30% (11/39) of cells (Figure 4A). Such abnormal polar localization was observed in less than 1% (2/201) of cells in the same strain without azide. When the isogenic azide-resistant *secA204* mutant strain (MQ585) was grown in the presence of sodium azide, less than 1% (1/132) of the cells showed abnormal polar localization of MukB-GFP_uv4_ foci. This indicated that the SecA protein is the functional target protein of 1 mM sodium azide among many essential ATPases in *E. coli*.

We next asked if inhibition of SecA with sodium azide affects the localization of SeqA protein. SeqA binds to hemimethylated GATC sequences of newly synthesized DNA immediately after the passage of replication forks that are not yet fully methylated (Lu et al., 1994), allowing the use of SeqA to detect replication forks. Interestingly, treatment of the SeqA-GFP_uv4_-expressing strain (MQ190) with 1 mM sodium azide for 1 h in medium C at 30°C led to a strong fluorescence signal from abnormally large clusters of SeqA-GFP_uv4_ in approximately 40% (7/18) of the cells (Figure 4B). This may reflect the defect in partitioning of SeqA-GFP_uv4_ foci. These abnormal types of cells were seen in less than 5% (2/43) of cells in a control subculture without azide, which is consistent with the results from Onogi et al. (1999). The isogenic azide-resistant *secA204* strain (MQ337) exhibited fewer abnormalities (2/85), even in the presence of 1 mM sodium azide. These results show that inhibition of the SecA ATPase activity with sodium azide causes defects in the positioning of MukB-GFP_uv4_ and SeqA-GFP_uv4_ foci. This is in line with our assumption that SecA is involved in proper positioning of MukB and SeqA by uncharacterized mechanism.

### FRAP assay for SecA-GFP_UV4_, SecY-GFP_UV4_, AcpP-GFP_UV4_, and other fluorescent proteins

**Figure 5:**
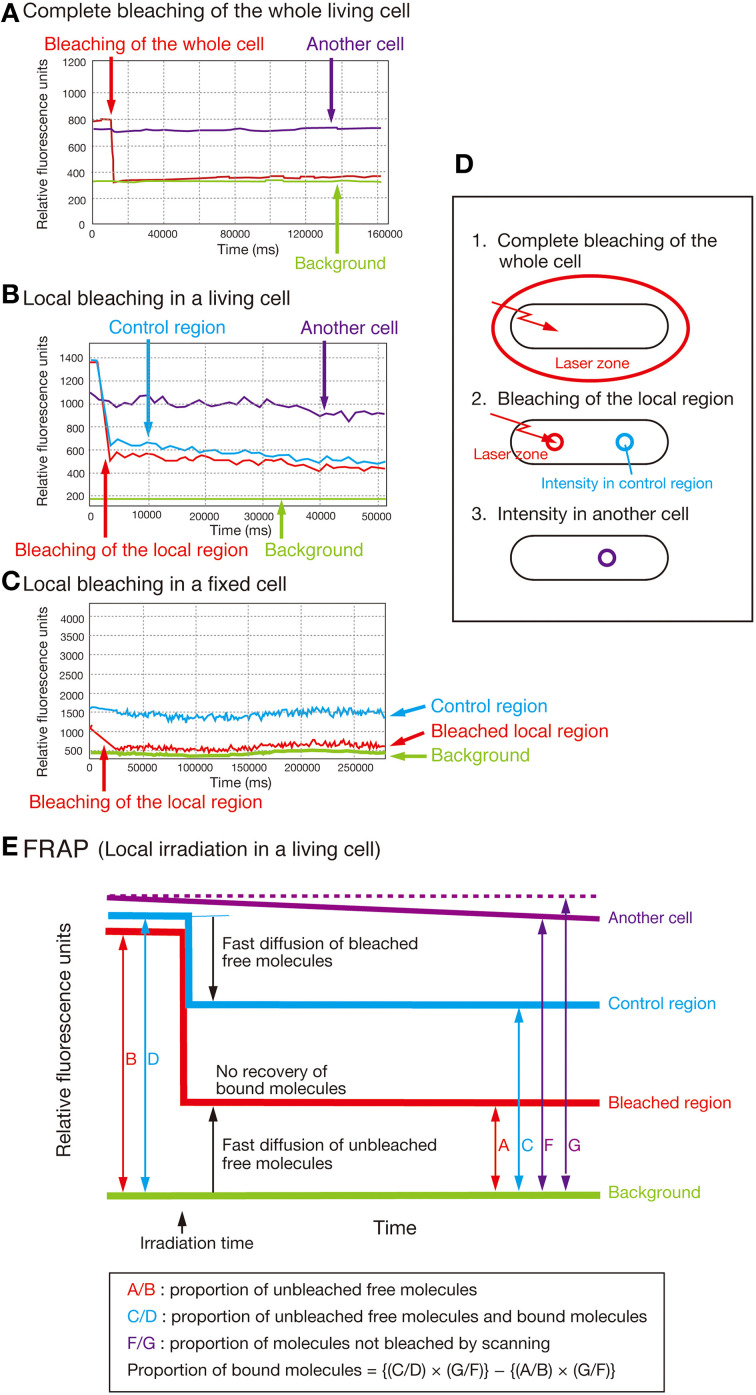
**FRAP analysis. (A)** Complete bleaching of the entire cell in the strain expressing SecA-GFP_uv4_ (MQ318). “Another cell” indicates another cell in exactly the same field that was not photobleached. **(B)** Typical FRAP data obtained with local bleaching in a cell of strain MQ318. **(C)** Effect of bleaching on the cells fixed with 1% formaldehyde in the strain MQ318 expressing SecA-GFP_uv4_. Note that the bleaching time was longer in fixed cells than in living cells. This is due to the reduced bleaching efficiency in fixed cells. **(D,E)** Concepts of the FRAP analysis.

To gain further insight into the biochemical mechanisms underlying the interdependency of SecA, SecY, and AcpP, we performed a FRAP assay (Axelrod et al., 1976) and quantified the proportion of the immobile form of SecA-GFP_uv4_, SecY-GFP_uv4_, and AcpP-GFP_uv4_ in living cells (Figure 5; for the control experiments, see Supplementary Text).

The results of the FRAP assay are summarized in Table 2. The average proportion of the immobile form of SecA-GFP_uv4_ was 5–10% in wild-type cells (MQ318) but was increased to 15–20% in the presence of 1 mM sodium azide (Exp. 1). Similarly, the immobile form increased to 15–20% both in the cold-sensitive *secY39*(Cs) (a stimulator of the SecA ATPase activity) mutant and the temperature-sensitive *acpP*D38V mutant at the non-permissive temperatures of 22 and 42°C, respectively (Exps. 2 and 3). These results are consistent with the observation that the dynamic localization of SecA-GFP_uv4_ was affected in these conditions.

**Table 2:** **FRAP analysis in various strains**.

**Exps**.	**Strain**	**Genotype**	**Temp**.	**Number of samples**	**Dynamics**	**Immobile form**	***p*-Value**
**SecA-GFP_uv4_**							
1	MQ318	WT	30°C	6	+	5.6 ± 4.4%	DP
		WT (+Az)	30°C	10	−	^*^16.0 ± 8.3%	0.025
2	MQ519	WT	22°C	10	+	9.2 ± 8.9%	0.19
	MQ435	*secY39* (Cs)	37°C	10	+	9.2 ± 8.8%	0.19
			22°C	10	−	^*^18.6 ± 12.9%	DP
3	MQ516	WT	42°C	10	+	6.7 ± 4.0%	0.079
	MQ456	*acpP*D38V	30°C	5	+	12.2 ± 4.9%	0.32
			42°C	10	−	^*^18.7 ± 13.5%	DP
4	MQ513	WT	30°C	6	+	11.9 ± 11.2%	DP
	MQ515	Δ*secB*	30°C	10	+	10.5 ± 8.5%	0.81
**SecY-GFP_uv4_**							
5	MQ319	WT	30°C	10	+	8.2 ± 8.2%	DP
		WT (+Az)	30°C	10	,	^*^13.7 ± 9.4%	0.33
6	MQ625	*secA204*	30°C	9	,	7.1 ± 7.5%	0.82
		*secA204* (+Az)	30°C	10	,	7.8 ± 6.4%	0.87
**AcpP-GFP_uv4_**							
7	MQ322	WT	30°C	10	,	3.3 ± 3.4%	DP
		WT (+Az)	30°C	8	,	3.0 ± 3.0%	0.88
**ParC-GFP_uv4_**							
8	MQ537	WT	30°C	10	+	3.9 ± 2.4%	DP
**ParE-GFP_uv4_**							
9	MQ539	WT	30°C	10	+	6.3 ± 3.1%	DP
**GyrA-GFP_uv4_**							
10	MQ323	WT	30°C	6	+	15.7 ± 7.0%	DP
**GyrB-GFP_uv4_**							
11	MQ324	WT	30°C	10	+	8.4 ± 3.6%	DP

WT represents the isogenic wild-type strain corresponding to each mutant. The secY39 mutation confers cold-sensitive growth (Cs) at 22°C. The secA204 mutation confers resistance to 4 mM sodium azide. (+Az) means that the experiments were done in the presence of 1 mM sodium azide. The errors are 99% confidence intervals from the Student's t-test estimation. The “p-value” is from Welch's test, whose data points are indicated as “DP.”

* Remarkable increase in the immobile form.

Note that the microscopy used in FRAP is entirely different from that used in time-lapse microscopy. For the FRAP assay, we imaged the cells with a sufficiently long exposure (~1 s) to ensure that all the signals could be detected in each image. On the other hand, for the time-lapse microscopy, we used a shorter exposure (~100 ms) for observing dynamic properties. Deletion of the protein encoded by *secB*, which is another stimulator of SecY ATPase, had no effect on the proportion of the immobile form of SecA-GFP_uv4_ (Exp. 4).

The average proportion of the immobile form of SecY-GFP_uv4_ was 7–8% in wild-type cells and 14% in the presence of 1 mM sodium azide (Exp. 5). On the other hand, addition of sodium azide had no effect on the immobile form of SecY-GFP_uv4_ in the azide-resistant *secA204* mutant (Exp. 6). The discrepancy between the FRAP data and the time-lapse data might be due to the different microscopic systems to detect the fluorescence. Further explanation is described in the Discussion section. In contrast, addition of sodium azide had no remarkable effect on the proportion of the immobile form of AcpP-GFP_uv4_ (Exp. 7). For ParC-GFP_uv4_, ParE-GFP_uv4_ and GyrB-GFP_uv4_, the proportions of immobile molecules were 5–10%, similar to the other molecules studied (Exps. 8, 9, 11). For GyrA-GFP_uv4_, the proportion of immobile molecule was ~15%, slightly higher than the other molecules (Exp. 10). This might be the consequence of GyrA functions acting as a hub of protein–protein interaction network, involving MreB and PstB that interact with inner cell membrane proteins (Butland et al., 2005). For MukB, we were unable to bleach the small strong fluorescent foci without the effect on other regions with lower fluorescence because of the failure in making a small bleaching region in our system. Additionally, in wild-type strains, the proportion of the immobile form of SecA-GFP_uv4_ was the same at 37, 30, and 22°C, showing that the various temperatures did not affect the dynamics detected with FRAP.

## Discussion

To briefly summarize, the present work demonstrates: (I) SecA, SecY, and AcpP localizations were non-uniform with condensed regions, embracing nucleoids; (II) SecA and SecY, together with MukB, Topo IV, and DNA gyrase, were dynamically localized in living cells; (III) Perturbation of SecY and AcpP resulted in abolishment of the dynamic localization of SecA; (IV) Conversely, perturbation of SecA results in abolishment of the dynamic localization of SecY; (V) The SecA ATPase activity was essential for the dynamic localization of SecY.

Chemical inhibition using 1 mM sodium azide (Figure 2I) or conditional genetic inhibition (Figure 2H) of SecA abolished the dynamic localization of SecY. These results indicate that SecA plays an essential role in the dynamics and that the rapid change in local patterns of these proteins is not due to random Brownian motion of protein molecules. Conversely, SecY or AcpP deficiency perturbed the normal dynamics of SecA (Figures 2C,E). These data suggest that SecA, SecY, and AcpP function in an interdependent manner and that the dynamics observed are not secondary effect of the mechanisms based on the other proteins, such as the secondary effects caused by aberrant membrane translocation of other proteins or proteins that localize dynamically besides SecA/SecY/AcpP. The results from the FRAP assay (Table 2) indicated that the addition of sodium azide caused an increase in the proportion of the immobile form (possibly the tortuous multimer along nucleoids, see Figure 1A) of SecA-GFP_uv4_ and that the immobile form of SecA-GFP_uv4_ was dependent on the ATP-bound immobile form of SecA (van der Wolk et al., 1997; Bauer et al., 2014) (Table 2, Exp. 1). Note that SecA exists both in peripheral of inner cell membrane and cytoplasm (Cabelli et al., 1991). SecA-ATP may be in an active polymeric state and form a stable bridge for chromosomes in this situation, as mentioned in Introduction section. SecY stimulates the ATPase activity of SecA, and therefore, SecY may stimulate release of the SecA bridge from the nucleoid by converting immobile SecA-ATP to mobile SecA-ADP (Bauer et al., 2014). In agreement with this speculation, the *secY*(Cs) mutation caused an increase in the proportion of the immobile form of SecA-GFP_uv4_ at the non-permissive temperature (Table 2, Exp. 2). SecB also stimulates the ATPase activity of SecA (Miller et al., 2002), but deletion of *secB* (MQ515) did not affect the proportion of the immobile form of SecA-GFP_uv4_ (Table 2, Exp. 4). Furthermore, the dynamic localizations of MukB, DNA gyrase and Topo IV are abolished in the presence of 1 mM sodium azide (our unpublished data). These results suggest that SecA/SecY, together with MukB and DNA topoisomerases, can act as a molecular “tether” for bacterial chromosomes by mediating the stressing (via the immobile form) and releasing (via the mobile form) cycles of chromosomes (Fisher et al., 2013). For further discussion of AcpP-GFP_uv4_, see the Supplementary Text.

As we briefly mentioned in the Results section, there is a discrepancy between the FRAP data and the time-lapse data: higher proportion (up to approximately 20%) of immobile forms in the mutants under non-permissive conditions or the azide treatment (defined as “disturbed conditions”) in FRAP data (Table 2), and reduced fluorescence under the disturbed conditions in the time-lapse imaging (Figures 2C,E,H). The discrepancy might be due to the different microscopic systems to detect the fluorescence. As one of the possible explanations, we speculate that the fluorescence molecules are classified into three forms: (1) immobile molecules centralized by SecA-ATP (Bauer et al., 2014); (2) molecules with medium values of diffusion coefficient; (3) molecules with the highest values of diffusion coefficient. The immobile form of SecA should be immobile SecA-ATP because in FRAP experiments, immobility is observed in the order of minutes, which is in agreement with “Push and Slide” mechanism proposed by Bauer et al. (2014). In the FRAP system (Table 2), the type 1 immobile molecules were 5–20% of the total fluorescence. The rest of fluorescence fraction might be classified into the two types, 2 and 3. On the other hand, in the time-lapse imaging, the type 3 molecules were unable to be detected because the diffusion speed of the type 3 molecules was too rapid. The major fraction of fluorescence detected in the time-lapse imaging of wild-type cells (Figures 2A,F) might be the type 2 molecules with medium values of diffusion coefficient, regarding that the fraction of type 1 molecule is less than 20%. In the mutants of time-lapse imaging under disturbed conditions (Figures 2C,E,H), the type 2 molecules might be reduced, while the type 3 molecules was remarkably increased, resulting in the intensive reduction of detectable fluorescence. As a whole, in the disturbed conditions, a large portion of the type 2 molecules might be converted to the type 3 molecules and a small portion might be converted to the type 1 molecules, resulting in the small increase of the type 1 molecules up to approximately 20% of fluorescence in FRAP. Fluorescence images of the time-lapse imaging thus resulted in the remarkable reduction of detectable fluorescence and disappearance of the dynamics of fluorescence localization. Therefore, the type 2 molecules act a prominent role on the observed dynamics in wild-type cells, which is performed by cooperative functions of SecA, SecY, and AcpP. The types 1, 2, and 3 of molecules presumably correspond to immobile core of the multimer interacting with other membrane proteins, dynamic multimer with medium values of diffusion coefficient, and monomer with the highest values of diffusion coefficient, respectively, similar to the case in eukaryotic actin filament.

Perturbation of the SecA ATPase by sodium azide disrupted the positioning of MukB-GFP_uv4_ and the proper organization of SeqA-GFP_uv4_. These results indicate that SecA/SecY/AcpP dynamics may be involved in the organization of MukB and SeqA. Furthermore, according to DNA decatenation and superhelicity data, SecA has epistasis over MukB and Topo IV/DNA gyrase (Adachi et al., 2014). These data are further supported by the protein–protein interactions. DNA-binding chromosome partitioning proteins such as MukB, Topo IV, and DNA gyrase appear to physically interact with SecA, SecY, and AcpP, among approximately 4300 proteins in *E. coli* (Butland et al., 2005). The results of the large-scale protein-protein interaction network suggest the presence of a sub-network of SecA-AcpP-MukB-ParC/ParE-GyrA/GyrB. In addition, AcpP co-purifies with MukB (Niki et al., 1992), and SeqA interacts with ParC (Kang et al., 2003). Stimulation of Topo IV activity by MukB and execution of MukB activity through the Topo IV-MukB interaction have been shown in previous studies (Hayama and Marians, 2010; Li et al., 2010; Hayama et al., 2013). Additionally, MukB is essential for the decatenation function of DNA gyrase and Topo IV *in vivo* (Adachi et al., 2014). According to the dynamic nature of the proteins, it may be possible that reaction-diffusion type positioning that is due to oscillating substances is achieved in the process, in which SecA is an activator and SecY is an inhibitor, similar to the bacterial Min system, which determines the plane of cell division (Rowlett and Margolin, 2013).

It may be possible that the observed deficiencies in dynamics of the proteins are secondary effects of inhibition of the membrane protein translocation. However, immediate inhibition of the dynamics (within 5 min) after the addition of 1 mM sodium azide or *secY39*(Cs) mutation suggests that this possibility is unlikely. *secY39*(Cs) mutant cells lose their translocation activity for membrane proteins within 1 min of being placed in a cold temperature (22°C) (Baba et al., 1990). In addition, wild-type cells with a normal *secA* allele grew exponentially at least for 2 h after the addition of the chemical compound, and then the growth was gradually inhibited, suggesting that growth defects due to abolishing protein translocation were not prominent during a time scale of minutes (Adachi et al., 2014). Furthermore, *secA204* azide-resistant mutant, in which the membrane protein translocation activity of SecA is normal, exhibited abnormal localization of the *oriC* loci and a mild defect in SecY-GFP_uv4_ dynamics even in the absence of sodium azide (Adachi et al., 2014, this work). These results indicate that SecA is involved in proper positioning mechanism of *oriC* independently of the membrane protein translocation activity.

The apparent correlation between the dynamic properties of SecA/SecY/AcpP and the organization of the chromosome partitioning proteins may be important for these proteins to act as the molecular bridge between the inner cell membrane and chromosomes, possibly through molecular “tether” activity described in Fisher et al. (2013). Here, we can speculate that the tip of translocating multiple transmembrane protein can be immediately folded and interact with other membrane proteins that are often tightly linked with immobile protein multimers or cytoskeltons. This may make SecA-ATP in immobile form whereas SecA-ADP is in mobile form. Here, the mechanical force of SecA-ATP can result in pulling force (Bauer et al., 2014) of SecA-interacting AcpP-MukB or DNA topoisomerases (Adachi et al., 2014). From this speculation one may think that SecA might be involved in chromosome partitioning of *E. coli*. The present data on dynamics show that SecA and SecY may mediate the shorter scale (second order) dynamics of sister chromosomes described by Fisher et al. (2013). That is, these proteins may accumulate and release intra-nucleoid stress as the global molecular tether between the cell membrane and nucleoids acting on radial confinement and dynamic nucleoid density spots. On the other hand, an unknown programmed tether acts on a 20-min scale and promotes disposition of *oriC*, *ter*, and various loci in sister nucleoids at particular cell stages. Deficiency in SecA results in alteration of DNA topology such as superhelicity and decatenation (Adachi et al., 2014). This may be due to the absence of mechanical “greasing” activity of tethers as described in Fisher et al. (2013), resulting in a defect in separation of sister chromosomes. Chromosomal partitioning defects were observed in *secA*, *secY*, and *acpP* mutants (Adachi et al., 2014).

Our current results show that the dynamics of the membrane protein translocation machinery may be linked to chromosome partitioning and/or chromosome structure maintenance, possibly via bridging between SecA/SecY and AcpP/MukB/DNA gyrase/Topo IV/SeqA.

### Conflict of interest statement

The authors declare that the research was conducted in the absence of any commercial or financial relationships that could be construed as a potential conflict of interest.

## Supplementary material

Movies S1–S24. Z-stacked deconvoluted images. For details, see the legend to Figure 1. Red, GFP/native immunofluorescnece. Green, DAPI staining.

Movie S1 **Nucleoid with tortuous structures (PA340)**.

Movie S2 **SecA-GFP_uv4_ with tortuous localization (MQ318)**.

Movie S3 **Nucleoid with tortuous localization (MQ318)**.

Movie S4 **Merged movie with tortuous localization (MQ318)**.

Movie S5 **SecY-GFP_uv4_ with tortuous localization (MQ319)**.

Movie S6 **Nucleoid with tortuous localization (MQ319)**.

Movie S7 **Merged movie with tortuous localization (MQ319)**.

Movie S8 **AcpP-GFP_uv4_ with tortuous localization (MQ322)**.

Movie S9 **Nucleoid with tortuous localization (MQ322)**.

Movie S10 **Merged movie with tortuous localization (MQ322)**.

Movie S11 **GFP_uv4_ (MQ668)**.

Movie S12 **Nucleoid with tortuous localization (MQ668)**.

Movie S13 **Merged movie with tortuous localization (MQ668)**.

Movie S14 **A control for GFP_uv4_ (non-fusion) staining (PA340)**.

Movie S15 **Nucleoid with tortuous localization (PA340)**.

Movie S16 **SecA with tortuous localization (PA340)**.

Movie S17 **Nucleoid with tortuous localization (PA340)**.

Movie S18 **Merged movie of S16 and S17 with tortuous localization (PA340)**.

Movie S19 **SecY with tortuous localization (PA340)**.

Movie S20 **Nucleoid with tortuous localization (PA340)**.

Movie S21 **Merged movie of S19 and S20 with tortuous localization (PA340)**.

Movie S22 **AcpP with tortuous localization (PA340)**.

Movie S23 **Nucleoid with tortuous localization (PA340)**.

Movie S24 **Merged movie of S22 and S23 with tortuous localization (PA340)**.

Movies S25–S40. Time-lapse movies of GFP_**uv4**_-fused proteins. Time-lapse images were taken by exposure for 100 ms, and the interval of each frame is 3 s. For details, see legend to Figure 2.

Movie S25 **SecA-GFP_**uv4**_ in wild-type cells (MQ318) growing at 30°C**.

Movie S26 **SecA-GFP_**uv4**_ in *secY39*(Cs) mutant cells (MQ435) growing at the permissive temperature of 37°C**.

Movie S27 **SecA-GFP_**uv4**_ in *secY39*(Cs) mutant cells (MQ435) growing at the non-permissive temperature of 22°C for 5 min**.

Movie S28 **SecA-GFP_**uv4**_ in *acpP*D38V(Ts) mutant cells (MQ456) growing at the permissive temperature of 30°C**.

Movie S29 **SecA-GFP_**uv4**_ in *acpP*D38V(Ts) mutant cells (MQ456) growing at the non-permissive temperature of 42°C for 3 h**.

Movie S30 **SecY-GFP_**uv4**_ in wild-type cells (MQ319) growing at 30°C**.

Movie S31 **SecY-GFP_**uv4**_ in *secA51*(Ts) mutant cells (MQ748) growing at the permissive temperature of 30°C**.

Movie S32 **SecY-GFP_**uv4**_ in *secA51*(Ts) mutant cells (MQ748) growing at the non-permissive temperature of 42°C for 2 h**.

Movie S33 **SecY-GFP_**uv4**_ in wild-type cells (MQ319) growing at 30°C in the presence of 1 mM sodium azide. The incubation time with azide was approximately 5 min**.

Movie S34 **SecY-GFP_**uv4**_ in *secA204* mutant cells (MQ625) growing at 30°C (without sodium azide)**.

Movie S35 **ParC-GFP_**uv4**_ in wild-type cells (MQ537) growing at 30°C**.

Movie S36 **ParE-GFP_**uv4**_ in wild-type cells (MQ539) growing at 30°C**.

Movie S37 **GyrA-GFP_**uv4**_ in wild-type cells (MQ323) growing at 30°C**.

Movie S38 **GyrB-GFP_**uv4**_ in wild-type cells (MQ324) growing at 30°C**.

Movie S39 **MukB-GFP_**uv4**_ in wild-type cells (MQ529) growing at 30°C**.

Movie S40 **GFP_**uv4**_ in wild-type cells (MQ668) growing at 30°C**.
