# Corrigendum: Dynamic nature of SecA and Its associated proteins in *Escherichia coli*

**DOI:** 10.3389/fmicb.2020.595994

**Published:** 2020-11-25

**Authors:** Shun Adachi, Yasuhiro Murakawa, Sota Hiraga

**Affiliations:** Department of Radiation Genetics, Graduate School of Medicine, Kyoto University, Kyoto, Japan

**Keywords:** chromosome partition, SecA, SecY, AcpP, MukB, DNA topoisomerase

In the original article, there was a mistake in Figure 2 as published. Panel J in the original figure was incorrectly labeled “SecA-GFP secA204.” Panel J in the corrected figure appearing below is now labeled “SecY-GFP secA204” in agreement with panels F to I presented in this section of the figure.

**Figure 2:**
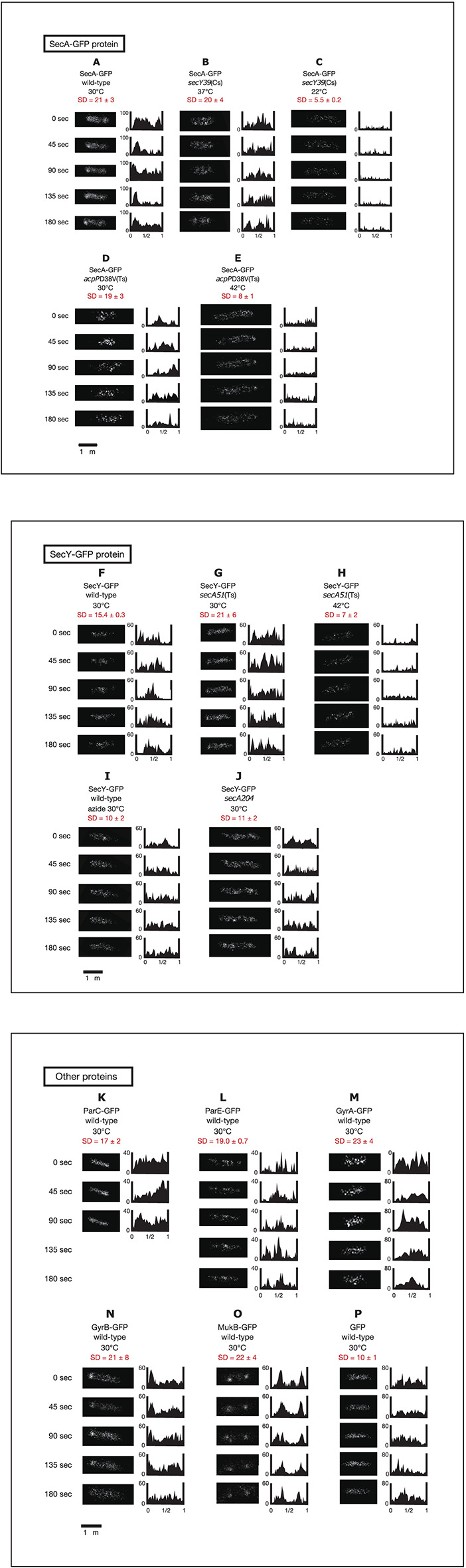
Time-lapse images of GFP_uv4_-fused proteins reveal the dynamic nature of chromosome partitioning proteins. Cells were grown in medium C and incubated with 0.1 mM IPTG for 1 h. Time-lapse images of fluorescence were taken by exposure for 100 ms. Both images and histograms of signals along long cell axes were obtained at 45-s intervals. The x-axes of histograms are subcellular positions and the y-axes of histograms are relative fluorescence units. Ninety-five Percent confidential SD (standard deviation) values of fluorescence signals along long cell axes in individual cells are also shown. **(A)** SecA-GFP_uv4_ in wild-type cells (MQ318) growing at 30°C. **(B)** SecA-GFP_uv4_ in *secY39*(Cs) mutant cells (MQ435) growing at the permissive temperature of 37°C. **(C)** SecA-GFP_uv4_ in *secY39*(Cs) mutant cells (MQ435) growing at the non-permissive temperature of 22°C. **(D)** SecA-GFP_uv4_ in *acpP*D38V(Ts) mutant cells (MQ456) growing at the permissive temperature of 30°C. **(E)** SecA-GFP_uv4_ in *acpP*D38V(Ts) mutant cells (MQ456) growing at the non-permissive temperature of 42°C. **(F)** SecY-GFP_uv4_ in wild-type cells (MQ319) growing at 30°C. **(G)** SecY-GFP_uv4_ in *secA51*(Ts) mutant cells (MQ748) growing at the permissive temperature of 30°C. **(H)** SecY-GFP_uv4_ in *secA51*(Ts) mutant cells (MQ748) growing at the non-permissive temperature of 42°C. **(I)** SecY-GFP_uv4_ in wild-type cells (MQ319) with 1 mM sodium azide growing at 30°C. The incubation time with azide was approximately 5 min. **(J)** SecY-GFP_uv4_ in *secA204* azide-resistant mutant cells (MQ625) growing at 30°C (without sodium azide). **(K)** ParC-GFP_uv4_ in wild-type cells (MQ537) growing at 30°C. **(L)** ParE-GFP_uv4_ in wild-type cells (MQ539) growing at 30°C. **(M)** GyrA-GFP_uv4_ in wild-type cells (MQ323) growing at 30°C. **(N)** GyrB-GFP_uv4_ in wild-type cells (MQ324) growing at 30°C. **(O)** MukB-GFP_uv4_ in wild-type cells (MQ529) growing at 30°C. **(P)** GFP_uv4_ in wild-type cells (MQ668) growing at 30°C.

The authors apologize for this error and state that this does not change the scientific conclusions of the article in any way. The original article has been updated.

