# Corrigendum: Dynamic nature of SecA and Its associated proteins in *Escherichia coli*

**DOI:** 10.3389/fmicb.2020.634250

**Published:** 2021-01-13

**Authors:** Shun Adachi, Yasuhiro Murakawa, Sota Hiraga

**Affiliations:** Department of Radiation Genetics, Graduate School of Medicine, Kyoto University, Kyoto, Japan

**Keywords:** chromosome partition, SecA, SecY, AcpP, MukB, DNA topoisomerase

In the original article, there were errors in Figures 1C,F and G as published. Images in the 1st and 2nd rows of Panel C and an image of DAPI in the 3rd row of Panel F are from different optical sections. For Panel G, the left column is from Native SecA, not Native SacA. The corrected Figure 1 appears below.

**Figure 1:**
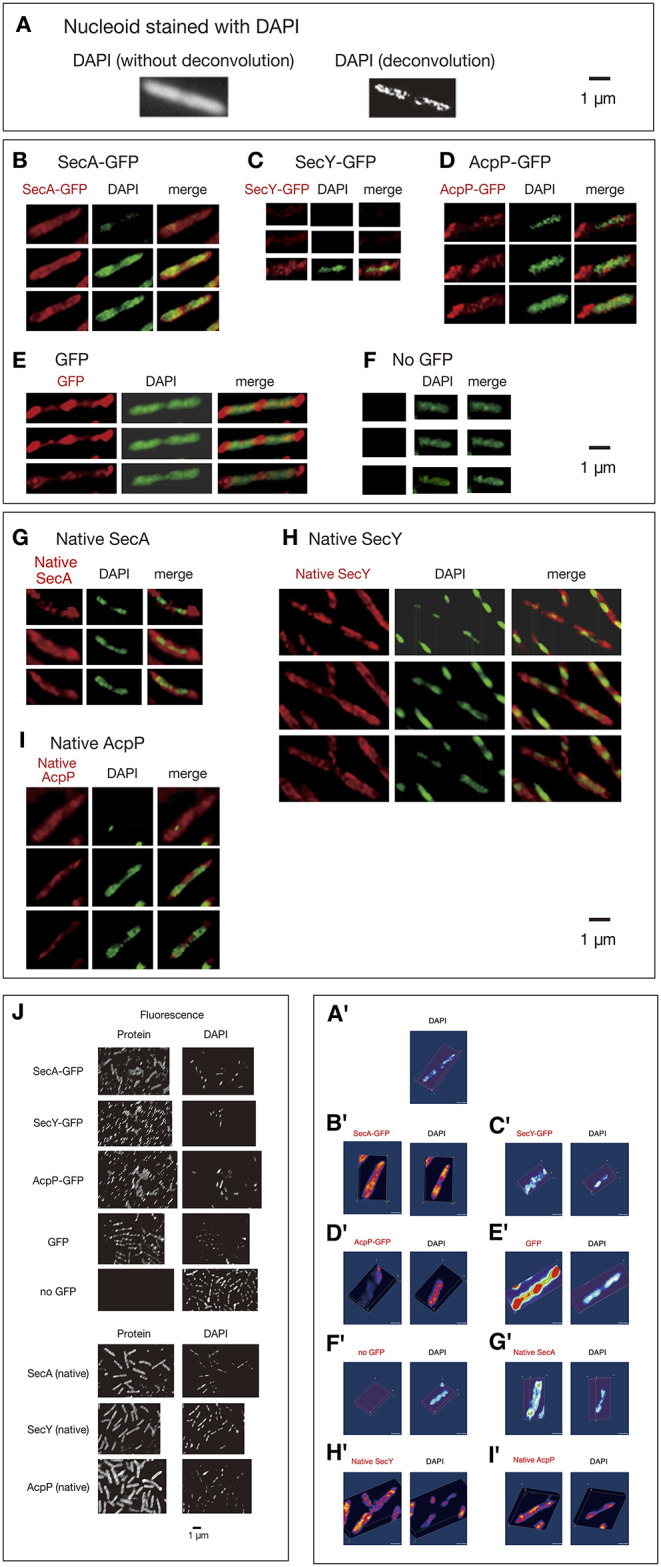
Three-dimensional deconvoluted images of various immunostained proteins. Cells were grown in medium L at 30°C and fixed with 70% methanol. Red, immunofluorescence of GFP or native proteins. Green, DAPI staining. Merge, merged images of red and green images. Note that synthesized yellow signal is only recognizable with eyes when the ratio of each signal is between ~4:6 and 6:4. More difference is recognizable as mere green or red. **(A)** Images of a DAPI-stained wild-type cell before and after 3D deconvolution in the Z-axis. **(B–F)** Fixed cells expressing GFP_uv4_-fused proteins or GFP_uv4_ protein (not fused with any protein) were immunostained using an anti-GFP monoclonal antibody. Fluorescence images of the proteins were analyzed with 3D deconvolution at 0.5-μm intervals in the Z-axis. Images of DAPI-stained nucleoids were also analyzed with 3D deconvolution. The yellow arrowheads indicate the clustered localization of protein molecules. **(B)** SecA-GFP_uv4_, **(C)** SecY-GFP_uv4_, **(D)** AcpP-GFP_uv4_, **(E)** GFP_uv4_ (not fused with any protein), **(F)** No GFP_uv4_. **(G–I)** Deconvoluted images of native proteins. Wild-type cells (PA340) were grown in medium L at 37°C, fixed, and immunostained using rabbit antisera for native proteins. Images of DAPI-stained nucleoids were also deconvoluted. **(G)** Native SecA, **(H)** Native SecY, **(I)** Native AcpP, **(J)** Three-dimensional deconvolution images of immunostained cells in wider sights corresponding to **(B)**–**(I)**. **(A'–I')** Three-dimensional images corresponding to **(A)**–**(I)**.

The authors apologize for this error and state that this does not change the scientific conclusions of the article in any way. The original article has been updated.

