# Supplementary material for: Dynamic nature of SecA and its associated proteins in Escherichia coli
Source: Front Microbiol. 2015 Feb 10;6:75. doi: 10.3389/fmicb.2015.00075 (PMC4322705; doi:10.3389/fmicb.2015.00075)
Supplement: Supplementary file 1 [file Table1.DOC]

**Table S1 | *Escherichia coli* strains used.**

-----------------------------------------------------------------------------------------------------------------------------------------------------------------------------------------------------

Strain Relevant genotype/description Resistance Reference/source

-----------------------------------------------------------------------------------------------------------------------------------------------------------------------------------------------------

W208 *thr-1 leuB6*(Am) *secA204 lacZ4 glnV44*(As) *rpsL8 thi-1* Sm-R, Az-R *E. coli* Genetic Stock Center

MC4100 *araD139* Δ(*argF-lac*)*U169 rpsL150 relA1 flbB5301 deoC1 ptsF25 rbsR* Sm-R Ito et al. (1981)

MM52 MC4100 except *secA51*(Ts) Sm-R Oliver and Beckwith (1981)

TYO MC4100 except *ompT*::*kan* *znd33*::Tn*10 rpsE* Km-R Tc-R Akiyama and Ito (1990)

AD208 TYO except *secY39* (Cs) Km-R Tc-R Baba et al. (1990)

NRD52 MG1655 except *ΔfabF::cat* Cm-R De Lay and Cronan (2006)

NRD29 NRD52 except *acpP* D38V Cm-R De Lay and Cronan (2006)

TL212 HfrC *phoA8 glpD3 glpR2 relA1 spoT1 pit-10 fhuA22 ompF627 fadL701* Tc-R Larson et al. (1984)

(λ) *glpK^i^ plsB26 gyrA* *zce-726*::*Tn*10

MQ442 NRD52 except *zce-726*::*Tn*10 *fabF*^+^ Tc-R, Cm-S This work

MQ441 NRD29 except *zce-726*::*Tn*10 *fabF*^+^ Tc-R, Cm-S This work

C600 *thi thr leuB lacY tonA supE* Appleyard (1954)

EJ812 C600 except *parC1215*(Ts) Kato et al. (1988)

W3110 IN (*rrnD-rrnE*) *1* *rph-1* Hill and Harnish (1981)

W3110 *parE10* W3110 except *parE10*(Ts) Kato et al. (1990)

YK1100 W3110 except *trpC9941* Yamanaka et al. (1996)

AZ5372 YK1100 except Δ*mukB*::*kan* Km-R Yamanaka et al. (1996)

KAT1 YK1100 except *mukB-gfpUV4 cat* Cm-R Ohsumi et al. (2001)

KK259 YK1100 except *seqA::tet* Tc-R Onogi et al. (2000)

HF4704 *polA thyA uvrA phx* Kreuzer and Cozzarelli (1979)

KNK453 HF4704 except *gyrA43*(Ts) Kreuzer and Cozzarelli (1979)

LE316 *metB argE ilv tna supE (?) rpsL lac gal xyl thi gyrB*(Ts) Sm-R Orr et al. (1979)

JC12334 *recF143 thi-1 his-4 proA2 argE3 thr-1 leuB6 ara-14 lacY1 galK2 xyl-5 mtl-1* Sm-R, Tc-R Lloyd et al. (1984)

*supE44 tsx-33 rpsL31* *tna-300*::*Tn*10

MQ574 LE316 except *tna-300*::*Tn*10 Tc-R This work

MQ583 HF4704 except *tna-300*::*Tn*10 Tc-R This work

MQ581 HF4704 except *tna-300*::*Tn*10 *gyrB*(Ts) Tc-R This work

BW25113 *lacI_q_ rrnBT14* Δ*lacZWJ16 hsdR514* Δ*araBADAH33,* Δ*rhaBADLD78*  Datsenko and Wanner (2000)

BΩ25113Δ*secB*::*kan* BW25113 except Δ*secB*::*kan* Km-R Baba et al. (2006)

PA340 *argH1 thr-1 leuB6 hisG1 ghd-1 gltB31 thi-1 lacY1 gal-6* Sm-R Wachi et al. (1989)

*xyl-7 ara-14 mtl-2 malA1 rpsL9 tonA2*

MQ329 PA340 except *secA204* Sm-R, Az-R This work

-------------------

Strains expressing GFP fusion proteins

MQ318 PA340 (pHis_6_-SecA-GFP_uv4_) Sm-R, Cm-R This work

MQ319 PA340 (pHis_6_-SecY-GFP_uv4_) Sm-R, Cm-R This work

MQ322 PA340 (pHis_6_-AcpP-GFP_uv4_) Sm-R, Cm-R This work

MQ529 PA340 *mukB-gfpUV4 cat* Sm-R, Cm-R This work

MQ190 PA340 (pHis_6_-SeqA-GFP_uv4_) Sm-R, Cm-R This work

MQ537 PA340 (pHis_6_-ParC-GFP_uv4_) Sm-R, Cm-R This work

MQ539 PA340 (pHis_6_-ParE-GFP_uv4_) Sm-R, Cm-R This work

MQ323 PA340 (pHis_6_-GyrA-GFP_uv4_) Sm-R, Cm-R This work

MQ324 PA340 (pHis_6_-GyrB-GFP_uv4_) Sm-R, Cm-R This work

MQ668 PA340 (pGFP_gcn4_) Sm-R, Ap-R This work

MQ625 PA340 *secA204* (pHis_6_-SecY-GFP_uv4_) Sm-R, Cm-R, Az-R This work

MQ626 PA340 *secA204* (pHis_6_-AcpP-GFP_uv4_) Sm-R, Cm-R, Az-R This work

MQ585 PA340 *secA204 mukB-gfpUV4 cat* Sm-R, Cm-R, Az-R This work

MQ337 PA340 *secA204* (pHis_6_-SeqA-GFP_uv4_) Sm-R, Cm-R, Az-R This work

MQ630 PA340 *secA204* (pHis_6_-ParC -GFP_uv4_) Sm-R, Cm-R, Az-R This work

MQ631 PA340 *secA204* (pHis_6_-ParE-GFP_uv4_) Sm-R, Cm-R, Az-R This work

MQ628 PA340 *secA204* (pHis_6_-GyrA-GFP_uv4_) Sm-R, Cm-R, Az-R This work

MQ629 PA340 *secA204* (pHis_6_-GyrB-GFP_uv4_) Sm-R, Cm-R, Az-R This work

MQ743 MC4100 (pHis_6_-SecY-GFP_uv4_) Cm-R, Sm-R This work

MQ748 MM52 (pHis_6_-SecY-GFP_uv4_) Cm-R, Sm-R This work

MQ519 TYO (pHis_6_-SecA-GFP_uv4_) Km-R, Tc-R, Cm-R This work

MQ435 AD208 (pHis_6_-SecA-GFP_uv4_) Km-R, Tc-R, Cm-R This work

MQ516 MQ442 (pHis_6_-SecA-GFP_uv4_) Tc-R, Cm-R This work

MQ456 MQ441 (pHis_6_-SecA-GFP_uv4_) Tc-R, Cm-R This work

MQ557 C600 (pHis_6_-SeqA-GFP_uv4_) Cm-R This work

MQ563 EJ812 (pHis_6_-SeqA-GFP_uv4_) Cm-R This work

MQ513 BW25113 (pHis_6_-SecA-GFP_uv4_) Cm-R This work

MQ515 BW25113 Δ*secB*::*kan* (pHis_6_-SecA-GFP_uv4_) Km-R, Cm-R This work

MQ410 MM52 (pHis_6_-SecA-GFP_uv4_) Sm-R, Cm-R This work

MQ528 AD208 (pHis_6_-SecY-GFP_uv4_) Sm-R, Cm-R This work

MQ458 MQ441 (pHis_6_-AcpP-GFP_uv4_) Cm-R This work

MQ417 KK259 (pHis_6_-SeqA-GFP_uv4_) Tc-R, Cm-R This work

MQ561 EJ812 (pHis_6_-ParC-GFP_uv4_) Cm-R This work

MQ566 W3110 *parE10* (pHis_6_-ParE-GFP_uv4_) Cm-R This work

MQ649 KNK453 (pHis_6_-GyrA-GFP_uv4_) Cm-R This work

MQ644 MQ581 (pHis_6_-GyrB-GFP_uv4_) Tc-R, Cm-R This work

-----------------------------------------------------------------------------------------------------------------------------------------------------------------------------------------------------

**REFERENCES**

Akiyama, Y., and Ito, K. (1990). SecY protein, a membrane embedded secretion factor of *E. coli*, is cleaved by the OmpT protease *in vitro*. *Biochem. Biophys. Res. Commun.* 167, 711-715.

Appleyard, R.K. (1954). Segregation of new lysogenic types during growth of a doubly lysogenic strain derived from *Escherichia coli* K12. *Genetics* 39, 440-452.

Baba, T., Ara, T., Hasegawa, M., Takai, Y., Okumura, Y., Baba, M., et al. (2006). Construction of *Escherichia coli* K-12 in-frame, single-gene knockout mutants: the Keio collection. *Mol. Systems Biol.* doi:10.1038/msb4100050.

Baba, T., Jacq, A., Brickman, E., Beckwith, J., Taura, T., Ueguchi, C., et al. (1990). Characterization of cold-sensitive *secY* mutants of *Escherichia coli*. *J. Bacteriol.* 172, 7005-7010.

Datsenko, K.A., and Wanner, B.L. (2000). One-step inactivation of chromosomal genes in *Escherichia coli* K-12 using PCR products. *Proc. Natl. Acad. Sci. USA.* 97, 6640-6645.

De Lay, N.R., and Cronan, J.E. (2006). Gene-specific random mutagenesis of *Escherichia coli in vivo*: isolation of temperature-sensitive mutations in the acyl carrier protein of fatty acid synthesis. *J. Bacteriol.* 188, 287-296.

Hill, C.W., and Harnish, B.W. (1981). Inversion between ribosomal RNA genes of *Escherichia coli*. *Proc. Natl. Acad. Sci. USA* 78, 7069-7072.

Ito, K., Bassford, P., and Beckwith, J. (1981). Protein localization in *E. coli*: is there a common step in the secretion of periplasmic and outer membrane proteins? *Cell* 24, 707-717.

Kato, J.I., Nishimura, R., Imamura, H., Niki, H., Hiraga, S., and Suzuki, H. (1990). New topoisomerase essential for chromosome segragation in *E. coli*. *Cell* 63: 393-404.

Kato, J.I., Nishimura, Y., Yamada, M., Suzuki, H., and Hirota, Y. (1988). Gene organization in the region containing a new gene involved in chromosome partition in *Escherichia coli*. *J. Bacteriol.* 170, 3967-3977.

Kreuzer, K.N., and Cozzarelli, N.R. (1979). *Escherichia coli* mutants thermosensitive for deoxyribonucleic acid gyrase subunit A: effects on deoxyribonucleic acid replication, transcription, and bacteriophage growth. *J. Bacteriol.* 140, 424-435.

Larson, T.J., Ludtke, D.N., and Bell, R.M. (1984). sn-Glycerol-3-phosphate auxotrophy of *plsB* strains of *Escherichia coli*: evidence that a second mutation, *plsX*, is required. *J. Bacteriol.* 160, 711-717.

Lloyd, R.G., Benson, F.E., and Shurvinton, C.E. (1984). Effect of *ruv* mutations on recombination and DNA repair in *Escherichia coli* K12. *Mol. Gen. Genet.* 194, 303-309.

Ohsumi, K., Yamazoe, M., and Hiraga, S. (2001). Different localization of SeqA-bound nascent DNA clusters and MukF-MukE-MukB complex in *Escherichia coli* cells. *Mol. Microbiol.* 40, 835-845.

Oliver, D., and Beckwith, J. (1981). *E. coli* mutant pleiotropically defective in the export of secreted proteins. *Cell* 25, 765-772.

Onogi, T., Yamazoe, M., Ichinose, C., Niki, H., and Hiraga, S. (2000). Null mutation of the *dam* or *seqA* gene suppresses temperature-sensitive lethality but not hypersensitivity to novobiocin of *mukB* null mutants. *J. Bacteriol.* 182, 5898-5901.

Orr, E., Fairweather, N.F., Holland, I.B., and Pritchard, R.H. (1979). Isolation and characterization of a strain carrying a conditional lethal mutation in the *cou* gene of *Escherichia coli* K12. *Mol. Gen. Genet.* 177, 103-112.

Wachi, M., Doi, M., Okada, Y., and Matsuhashi, M., (1989). New *mre* genes *mreC* and *mreD*, responsible for formation of the rod shape of *Escherichia coli* cells. *J. Bacteriol.* 171, 6511-6516.

Yamanaka, K., Ogura, T., Niki, H., and Hiraga, S. (1996). Identification of two new genes, *mukE* and *mukF*, involved in chromosome partitioning in *Escherichia coli*. *Mol. Gen. Genet.* 250, 241-251.
