# Supplementary material for: Dynamic nature of SecA and its associated proteins in Escherichia coli
Source: Front Microbiol. 2015 Feb 10;6:75. doi: 10.3389/fmicb.2015.00075 (PMC4322705; doi:10.3389/fmicb.2015.00075)
Supplement: Supplementary file 2 [file Table2.DOC]

**Table S2 | Plasmids used.**

--------------------------------------------------------------------------------------------------------------------------------------------------------------------------------------------------------------------------------

Plasmid Replicon Relevant genotype/description Resistance Reference/source

--------------------------------------------------------------------------------------------------------------------------------------------------------------------------------------------------------------------------------

pGFP_gcn4_ pMB1 p*lac::*GFP_uv4_ Ap-R Ito et al. (1999)

pHis_6_-SecA-GFP_uv4_ pR6K pT5-*lac::* *his_6_::secA::gfp_gcn4_* Cm-R Kitagawa et al. (2005)

pHis_6_-SecY-GFP_uv4_ pR6K pT5-*lac::* *his_6_::secY:: gfp_gcn_* Cm-R Kitagawa et al. (2005)

pHis_6_-AcpP -GFP_uv4_ pR6K pT5-*lac::* *his_6_::acpP:: gfp_gcn_* Cm-R Kitagawa et al. (2005)

pHis_6_-SeqA-GFP_uv4_ pR6K pT5-*lac::* *his_6_::seqA:: gfp_gcn_* Cm-R Kitagawa et al. (2005)

pHis_6_-ParC -GFP_uv4_ pR6K pT5-*lac::* *his_6_::parC:: gfp_gcn_* Cm-R Kitagawa et al. (2005)

pHis_6_-ParE-GFP_uv4_ pR6K pT5-*lac::* *his_6_::parE:: gfp_gcn_* Cm-R Kitagawa et al. (2005)

pHis_6_-GyrA-GFP_uv4_ pR6K pT5-*lac::* *his_6_::gyrA:: gfp_gcn_* Cm-R Kitagawa et al. (2005)

pHis_6_-GyrB-GFP_uv4_ pR6K pT5-*lac::* *his_6_::gyrB:: gfp_gcn_* Cm-R Kitagawa et al. (2005)

-------------------------------------------------------------------------------------------------------------------------------------------------------------------------------------------------------------------------------

**REFERENCES**

Ito, Y., Suzuki, M., Husimi, Y. (1999). A novel mutant of green fluorescent protein with enhanced sensitivity for microanalysis at 488 nm excitation. *Biochem. Biophys. Res. Commun.* 264, 556-560.

Kitagawa, M., Ara, T., Arifuzzaman, M., Ioka-Nakamichi, T., Inamoto, E., Toyonaga, H., et al. (2005). Complete set of ORF clones of *Escherichia coli* ASKA library (A complete set of E. coli K-12 ORF archive): unique resources for biological research. *DNA Res.* 12, 291-299.
