# Supplementary material for: Dynamic nature of SecA and its associated proteins in Escherichia coli
Source: Front Microbiol. 2015 Feb 10;6:75. doi: 10.3389/fmicb.2015.00075 (PMC4322705; doi:10.3389/fmicb.2015.00075)
Supplement: Supplementary file 4 [file DataSheet1.DOC]

**SUPPLEMENTARY RESULT**

**CONTROL EXPERIMENTS FOR FRAP MICROSCOPY**

We first optimized the bleaching conditions by bleaching the whole cell (**Figure 5A**). The fluorescence signal from the cell of interest was completely diminished to the background level, whereas in other unbleached cells, the fluorescence signal did not change after ~1 second of bleaching and ~1 second of detection for each cell.

The typical experimental data for bleaching a local region (laser zone, ~0.1 µm^2^) within the cell are shown in **Figure 5B**. After bleaching a local region with a single pulse of the laser, the fluorescence intensity of the bleached region decreased to and remained at a level that was still substantially higher than the background level. This indicates that a high fraction of GFP-fused molecules were present that diffused very rapidly from the unbleached region (**Figure 5E**). This is a reasonable phenomenon because in this system, we used a detection period of ~1 second, which was substantially longer than a typical FRAP experiment. Taking into account that the typical diffusion constant of proteins in *E. coli* is 0.15 to 15 µm^2^/second (Elowitz et al., 1999; Meacci et al., 2006), modeling using the uniform circular disc profile (*D* = (0.88*w*^2^)/4*t*_1/2_; *D*; diffusion coefficient; *w*, radius of the disc, ~0.2 mm in this case) (Axelrod et al., 1976) gave a half recovery time (*t*_1/2_) of 0.0006 to 0.06 seconds, which was much shorter than the detection period of ~1 second. This shows the difficulty in measuring the diffusion coefficient in this system. We also modeled the possible fluorescence recovery rate with the following equation (Axelrod et al., 1976):

where *F*_K_(*t*) = normalized fluorescence intensity; *P* = plateau intensity; *K* = amount of bleaching; *t* = time, ~1 second (a single detection period); *t*_D_ = characteristic time = *w*^2^/4*D*; *w* = half-width at *e*^−2^ height of bleaching intensity, ~0.2 µm in this case. *K* can be calculated from the following equation (Axelrod et al., 1976) *in fixed cells only in this case* **(Figure 5C**):

*C_r_* = relative fluorescent intensity after bleaching, ~0.1 in this case; *r* = distance from bleaching center = *w* in this case. We estimated that 90–100% of the fluorescent recovery to the plateau value had already been achieved after a detection period of ~1 second, which is consistent with our results.

The fluorescence intensity of the control region within the same cell also decreased, indicating that the molecules in the bleached and unbleached control regions mixed due to fast diffusion. Using the equations *t*_D_ = *w*^2^/4*D* and *g*_D_ = *t*_1/2_/*t*_D_ ~2 at *K* ~17 (Axelrod et al., 1976), we estimated that a region of 1-100 µm^2^ in a restricted environment can be bleached to 50% fluorescent intensity during a bleaching period of ~1 second. About 50% fluorescent reduction was often seen in actual experiments of both bleached and unbleached regions. Because the sectional area of *E. coli* in medium C is ~1 µm^2^ (Adachi et al., 2006), we believe that the molecules in the bleached and unbleached control regions likely mixed due to rapid diffusion, resulting in only slight a difference from the immobile form of GFP fusion proteins.

Net bleaching also occurred with repeated scanning as observed in other unbleached cells (**Figure 5E**). We note that the bleaching pattern in our *E. coli* cells was different from the typical bleaching patterns obtained in eukaryotic cells. The difference was due to the long bleaching period and the weak signals from GFP-fused protein molecules compared with large eukaryotic cells, resulting in a requirement for much longer detection periods. In bacterial cells, a large proportion of diffusible molecules (tested proteins) are likely to pass through the laser zone very rapidly during the detection of the weak signals immediately after bleaching. Therefore, as mentioned earlier, we fixed the cells with 1% formaldehyde to see whether the diffusion effects were diminished, i.e., the presumed mixing of the fluorescent molecules between the two regions (bleached vs. control) disappeared. The fluorescence intensity of the bleached region decreased to the background level (**Figure 5C**). In contrast, the control region was not affected in the fixed cells. We thus concluded that the proportion of immobile molecules can be calculated with the formula shown in **Figure 5E**.

**SUPPLEMENTARY DISCUSSION**

Although the defect in AcpP caused an increase in the non-diffusible immobile form of SecA-GFP_uv4_ (**Table 2**, Exp. 3) and homogeneous distribution of SecA-GFP_uv4_ (**Figure 2E**), the proportion of non-diffusible immobile AcpP-GFP_uv4_ molecules was not affected in the presence of 1 mM sodium azide (**Table 2**, Exp. 7). This can be explained by the fact that a tremendous number of AcpP molecules (~5 × 10^4^ molecules per cell) (Niki et al., 1992) exists in the cell and that AcpP acts multifunctionally to acylate many molecules (Geiger and López-Lara, 2002). That is, the proportion of AcpP molecules that participates in the dynamic localization of the series of proteins may be small among the total number of AcpP molecules.

These assumptions also explain the result that dynamic localization of AcpP-GFP_uv4_ was not clear in our time-lapse experiments. The FRAP data furthermore suggest that the proportions of immobile SecA-GFP_uv4_, SecY-GFP_uv4_, and AcpP-GFP_uv4_ were not high; less than 10%. The large proportion of the mobile form of these proteins may enable their primary functions; membrane translocation of membrane proteins or acylation of many molecules.

**REFERENCES FOR SUPPLEMENTARY TEXT**

Adachi, S., Hori, K., and Hiraga, S. (2006). Subcellular positioning of F plasmid mediated by dynamic localization of SopA and SopB*. J. Mol. Bio*l. 356, 850-863.

Axelrod, A., Koppel, D.E., Schlessinger, J., Elson, E., and Webb, W.W. (1976). Mobility measurement by analysis of fluorescence photobleaching recovery kinetics. *Biophys. J.* 16, 1055-1069.

Elowitz, M.B., Surette, M.G., Wolf, P.E., Stock, J.B., and Leibler, S. (1999). Protein mobility in the cytoplasm of *Escherichia coli*. *J. Bacteriol.* 181, 197-203.

Geiger, O., and López-Lara, I.M. (2002). Rhizobial acyl carrier proteins and their roles in the formation of bacterial cell-surface components that are required for the development of nitrogen-fixing root nodules on legume hosts. *FEMS Microbiol. Lett.* 208, 153-162.

Meacci, G., Ries, J., Fischer-Friedrich, E., Kahya, N., Schwille, P., and Kruse, K. (2006). Mobility of Min-proteins in *Escherichia coli* measured by fluorescent correlation spectroscopy. *Phys. Biol.* 3, 255-263.

Niki, H., Imamura, R., Kitaoka, M., Yamanaka, K., Ogura, T., and Hiraga, S. (1992). *E. coli* MukB protein involved in chromosome partition forms a homodimer with a rod-and-hinge structure having DNA binding and ATP/GTP binding activities. *EMBO J.* 11, 5101-5109.
